# Improving the Accuracy of Papillary Thyroid Cancer Pathological Evaluation With Digital PCR Detection of the *BRAF* V600E Mutation

**DOI:** 10.1002/jcla.70323

**Published:** 2026-09-16

**Authors:** Na Yu, Kai Zhang, Ying Jiang, Liang Zheng, Xiu He, Qinglin He, Meng Dai, Tianhua Hu, Dongfang Tang, Jingjing Jin, Xin Huang, Li Xiao

**Affiliations:** ^1^ Department of Pathology Huadong Hospital, Fudan University Shanghai China; ^2^ Department of General Practice Huadong Hospital, Fudan University Shanghai China; ^3^ Mission Medical Technologies Yuyao Zhejiang Province China; ^4^ Zhejiang University‐University of Edinburgh Institute Hangzhou Zhejiang Province China; ^5^ Institute of Pathology University Medical Center Rostock Rostock Germany; ^6^ Department of General Surgery Huadong Hospital, Fudan University Shanghai China; ^7^ Department of Thoracic Surgery Huadong Hospital, Fudan University Shanghai China

**Keywords:** *BRAF* V600E, digital PCR, papillary thyroid cancer, pathological diagnosis

## Abstract

**Background:**

The *BRAF* V600E mutation is an important diagnostic factor for papillary thyroid cancer (PTC). Digital PCR (dPCR) is a relatively new technology featured with extremely high sensitivity. This study aims to evaluate the performance of dPCR in detecting *BRAF* V600E mutations in PTC diagnosis.

**Methods:**

A total of 252 thyroid samples were collected in this retrospective diagnostic accuracy study. Diagnosis was made by a pathologist following histopathological guidelines. The *BRAF* V600E mutation was detected by dPCR and the results were compared with calls from amplification refractory mutation system PCR (ARMS‐PCR) tests. Discrepant samples were evaluated again by two pathologists, and the presence of mutations was further confirmed by next generation sequencing (NGS). McNemar's test was used for paired categorical data in statistical analysis.

**Results:**

Test results from dPCR and ARMS‐PCR assays were in total agreement for PTC tumor and benign thyroid tissues. However, dPCR assays detected *BRAF* V600E mutations in four tumor‐adjacent normal tissues, one lymph node sample, and two fine‐needle aspirate (FNA) samples that were negative in ARMS‐PCR tests, all with low frequencies of *BRAF* V600E mutations. When pathology results were used as the standard, the sensitivity, specificity, positive predictive value (PPV), and negative predictive value (NPV) of dPCR assay were all 100% for tissue and FNA samples, while those of ARMS‐PCR assay were 91.2%, 100%, 100%, 96.1%, and 92.9%, 100%, 100%, 95.7% for tissue and FNA samples, respectively.

**Conclusions:**

Our data demonstrate that dPCR is a highly valuable tool for PTC molecular pathology.

## Graphical abstract

This study developed a highly sensitive and specific digital PCR assay for *BRAF* V600E mutation detection in papillary thyroid cancer (PTC). The increased sensitivity of *BRAF* mutation detection directly led to more accurate pathological diagnosis of PTC and in turn improved clinical care in patients with limited sample availability or in samples with extremely low tumor presence.

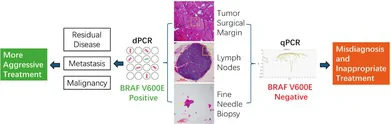

## 1 Introduction

According to GLOBOCAN 2022 estimate, 0.8 million thyroid cancer new cases and 47,000 deaths were estimated worldwide in 2022 [1]. Thyroid cancer has the third highest cancer incidence in China, with almost half million new cases and over 11,000 deaths in 2022 [2]. Both incidence and mortality of thyroid cancer had been on the rise, with an average annual percent change of 12.4% and 2.9% to incidence and mortality, respectively, from 2005 to 2015 [3].

PTC is the most common type of thyroid cancer, accounting for 80% to 85% of all thyroid cancer cases. For PTC diagnosis, ultrasound‐guided FNA cytopathology is a reliable, commonly used, and well accepted method [4]. However, up to one‐third of FNA specimens were classified into the indeterminate categories according to The Bethesda System for Reporting Thyroid Cytology (TBSRTC), leading to overdiagnosis or false‐negative diagnosis of thyroid cancer [5]. Molecular characterization of thyroid cancer has improved interpretation of the indeterminate cytology, of which the most common gene variant is the *BRAF* V600E mutation with a prevalence of 76.4% in East Asian PTC patients [6, 7]. The *BRAF* V600E mutation is also significantly associated with a high frequency of cervical lymph node metastases, locoregional recurrences, and less responsiveness to radioactive iodine [8]. Thus, accurate detection of the *BRAF* V600E mutation is of great importance in guiding PTC treatment strategy.

Surgical resection is the primary therapy for PTC treatment, followed by radioactive iodine therapy [6]. To accurately evaluate prognosis of PTC, it is essential to assess whether tumor disseminates within the glandular lobe or to lymph nodes for TNM staging [9]. Traditionally, formalin‐fixed paraffin‐embedded (FFPE) tissues of thyroids and regional lymph nodes are sectioned and evaluated by a pathologist to determine whether tumor cells have invaded any vessels, meaning that a large area of tissue section must be carefully evaluated under a microscope, a highly tedious process. Thus, it remains a challenge to thoroughly evaluate many tissue sections without missing a few cancerous cells. Even after rigorous evaluation, a definitive diagnosis of PTC still may not be reached for cases with atypical morphology, such as noninvasive follicular thyroid neoplasm with papillary‐like nuclear features (NIFTP) and PTC coexisting with chronic lymphocytic (Hashimoto) thyroiditis [10]. In these cases, the *BRAF* V600E mutation status becomes critical in helping reach a definitive diagnosis.

dPCR is a recently emerged PCR technology with extremely high sensitivity and is able to realize absolute quantification of nucleic acid targets [11]. In this study, we developed a dPCR assay for *BRAF* V600E mutation detection and assessed its application in pathological evaluation of PTC. Our data clearly demonstrate the value of highly sensitive *BRAF* V600E mutation detection in improving the accuracy of PTC pathology.

## 2 Materials and Methods

### 2.1 Patient Specimen Collection

This is a retrospective diagnostic accuracy study, 180 FFPE specimens from thyroidectomy, and 72 FNA biopsy samples with *BRAF* V600E testing results using ARMS‐PCR were collected from the Department of Pathology at Huadong Hospital between January 2024 and June 2025, including 57 PTC tumor tissues, 57 tumor‐adjacent thyroid tissues (≥ 1 cm away from the tumor), 4 lymph node samples from these PTC cases from the cervical central area, with suspected tumor metastasis, and 62 benign thyroid lesions with morphology similar to PTC. The 72 FNA biopsy samples were cytologically diagnosed as atypical cell/follicular lesions (TBSRTC III‐IV). All patients provided informed consent, and the study was approved by the Ethics Committee of Huadong Hospital (20240084).

Additional methods are included in the Supporting Informations.

## 3 Results

### 3.1 Characteristics of PTC Patients

We collected 118 paraffin samples from 57 PTC patients that had undergone ARMS‐PCR‐based *BRAF* V600E mutation tests (Table 1). Among the 57 PTC patients, 36 (63.2%) were females and 21 were males (36.8%). The mean age of patients was 47.5 ± 14.2 years (range from 18 to 79). 52 of the 57 PTC patients (91.2%) were positive for the *BRAF* V600E mutation.

**TABLE 1:** Characteristics of the PTC patients included in this study.

Characteristic	*N* = 57
Age (mean ± SD)	47.5 ± 14.2
Size (cm) (mean ± SD)	1.3 ± 0.6
Gender male	21 (36.8%)
Multifocality	10 (17.5%)
Extrathyroidal extension	4 (7.0%)
Lymph node metastasis	38 (66.7%)
*BRAF* positive	52 (91.2%)

### 3.2 Developing a Robust dPCR Assay for *BRAF* V600E Mutation Detection

To evaluate the analytical sensitivity of our dPCR assay, we serially diluted the DNA from RKO, a colorectal cancer cell line that harbors a *BRAF* V600E mutation, with DNA from PBMC from a healthy donor (ranging from 0.8% to 0.05%). Our dPCR assay was able to detect the *BRAF* V600E mutation at all dilutions, and the limit of detection (LOD) was determined to be 0.05% (Figure S1). Additionally, we calculated the coefficient of variation (CV) in various *BRAF* V600E mutation dilutions and the test results were highly reproducible, with a CV_0.8%_ = 2.41% and a CV_0.1%_ = 21.7%.

### 3.3 Detection of *BRAF* V600E Mutations in FFPE Samples

We collected 57 PTC samples and 57 cancer‐adjacent normal thyroid tissues. In complete agreement with the ARMS‐PCR test results, dPCR tests identified 52 PTC samples with *BRAF* V600E mutations, while five samples were *BRAF* WT (Table 2). However, among the 57 cancer‐adjacent normal thyroid tissues, dPCR assay detected four cases with *BRAF* V600E mutations, while ARMS‐PCR assay detected none (Tables 2 and 3). We investigated the reason for the discrepancy between ARMS‐PCR and dPCR assays by examining additional HE sections. Trace amount of PTC tumor cells was found in these discrepant samples (Figure 1A), with less than 20 tumor cells in each section. Notably, since total tissue areas of each section were approximately 1–1.5 cm^2^, with extremely low tumor proportions, sporadic tumor clusters could be easily missed under routine optical inspection or by less sensitive ARMS‐PCR assays. Indeed, all four cases displayed very low fractional abundance of the mutant allele (< 1.5%, Table 3).

**TABLE 2:** Summary of the *BRAF* V600E mutation test results using ARMS‐PCR and dPCR in FFPE samples.

Sample type	N	ARMS‐PCR^+^/dPCR^+^	ARMS‐PCR^+^/dPCR^−^	ARMS‐PCR^−^/dPCR^+^	ARMS‐PCR^−^/dPCR^−^
PTC tumor tissues	57	52	0	0	5
PTC tumor‐adjacent tissues	57	0	0	4	53
Benign thyroid lesions	62	0	0	0	62
Lymph nodes	4	0	0	1	3
Total	180	52	0	5	123

**TABLE 3:** Summary of the discrepant samples between ARMS‐PCR and dPCR in this study.

Patient ID	Mutation frequency in dPCR (copies/μL)	Postoperative pathology
11362	0.10% (5.56)	Intraglandular dissemination of PTC
13831	0.65% (51.184)	Intraglandular dissemination of PTC
15678	1.12% (92.136)	Intraglandular dissemination of PTC
17017	0.97% (31.159)	Intraglandular dissemination of PTC
15196	0.04% (4.472)	Lymph node, micro metastasis
00843	0.04% (34.32)	FNA, PTC (TBSRTC IV)
01021	0.02% (10.24)	FNA, PTC (TBSRTC IV)

**FIGURE 1:**
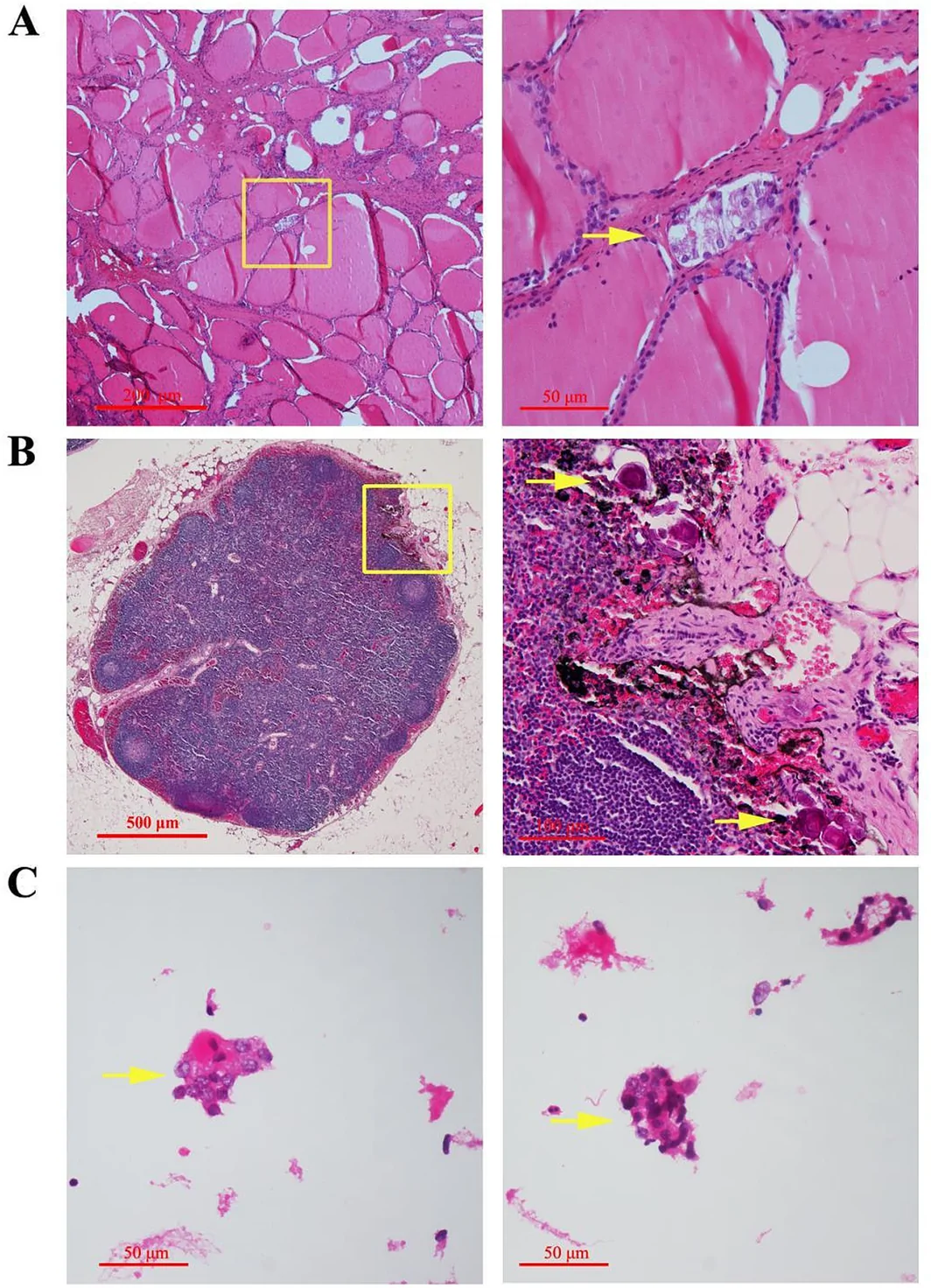
Histopathological morphology of samples with inconsistent results between dPCR and ARMS‐PCR assays. The right panels in A and B are the enlarged areas that were highlighted in a yellow square box from the left panels. The yellow arrows indicate lesions that are likely *BRAF* V600E positive. (A) Small tumor dissemination foci within thyroid tissue (4×/40×, bar = 200 μm/50 μm); (B) A small amount of gravel structure beneath the lymph node capsule in the central region of the neck, without metastatic tumor cells (2×/20×, bar = 500 μm/100 μm); (C) The FNA thyroid cytology samples (00843 at left panel and 01021 at right panel) evaluated as atypia of undetermined significance (TBSRTC‐III), which were later determined to be *BRAF* V600E positive by dPCR (40×, bar = 50 μm).

Four central lymph node samples from these PTC patients were examined for suspected tumor metastasis, including three patients with *BRAF* V600E positive tumors and one with *BRAF* V600E negative tumor. Despite deep sectioning and thorough assessment by two pathologists, the presence of metastatic epithelial cells was not found in HE and IHC (cytokeratin) sections, other than very few psammoma bodies in the subcapsular sinuses of lymph nodes (Figure 1B). No *BRAF* V600E mutation was detected in these lymph nodes by ARMS‐PCR. However, dPCR assay detected one sample with a *BRAF* V600E mutation (Table 2), with an extremely low allelic frequency of 0.04% (Table 3). A positive *BRAF* V600E mutation is sufficient for definitive diagnosis of lymph node metastasis for this patient, potentially changing the treatment strategy following surgery [12]. Our data demonstrated that highly sensitive dPCR testing improves the detection rate of *BRAF* V600E mutations in samples with extremely low tumor proportions. When pathology results were used as the standard, the sensitivity, specificity, PPV, and NPV of dPCR assay were all 100%, while those of ARMS‐PCR assay were 91.2% (52/57), 100% (123/123), 100% (52/52), and 96.1% (123/128), respectively (Table 4).

**TABLE 4:** The sensitivity, specificity, PPV, and NPV and their corresponding 95% confidence intervals (CIs) of ARMS‐PCR and dPCR assays in FFPE and FNA samples.

	FFPE		FNA	
	ARMS‐PCR	dPCR	ARMS‐PCR	dPCR
Sensitivity (95% CI)	91.2% (52/57) 81.0%–96.2%	100% (57/57) 93.7%–100%	92.9% (26/28) 77.4%–98.0%	100% (28/28) 87.9%–100%
Specificity (95% CI)	100% (123/123) 97.0%–100%	100% (123/123) 97.0%–100%	100% (44/44) 92.0%–100%	100% (44/44) 92.0%–100%
PPV (95% CI)	100% (52/52) 93.1%–100%	100% (57/57) 93.7%–100%	100% (26/26) 87.1%–100%	100% (28/28) 87.9%–100%
NPV (95% CI)	96.1% (123/128) 91.2%–98.3%	100% (123/123) 97%–100%	95.7% (44/46) 85.5%–98.8%	100% (44/44) 92%–100%

Follicular nodular disease accompanied by chronic lymphocytic is a common benign lesion in thyroid without genetic changes, but their atypical morphology and immunohistochemistry (IHC) results are difficult to distinguish from PTC. Consistent with literature, all 62 benign thyroid lesions were negative for the *BRAF* V600E mutation both in ARMS‐PCR and dPCR tests, indicating that our dPCR assay is highly specific.

### 3.4 Detection of *BRAF* V600E Mutations in FNA Specimens

*BRAF* V600E mutation test in FNA samples plays an important role in determining the nature of thyroid nodules, especially those with indeterminate or nondiagnostic cytology. A total of 72 indeterminate cytological samples of TBSRTC III‐V were examined by dPCR and ARMS‐PCR. ARMS‐PCR reported 26 FNA samples with *BRAF* V600E mutation (Table 5). However, dPCR detected two additional samples with a *BRAF* V600E mutation that were misclassified as WT by ARMS‐PCR (Table 3), indicating the malignant nature of these thyroid nodules (TBSRTC IV). These two patients were originally diagnosed as TBSRTC III, atypia of uncertain significance, in cytological smears (Figure 1C). Without further testing using dPCR, the negative results from ARMS‐PCR test would have led to misdiagnosis [13]. These 28 cytological samples were confirmed as *BRAF* V600E positive PTC in all follow‐up surgical specimens. The other 44 FNA samples were *BRAF* WT both in ARMS‐PCR and dPCR tests, with 42 diagnosed with TBSRTC III and two diagnosed with TBSRTC IV. These patients are undergoing regular follow‐up.

**TABLE 5:** Comparison of the *BRAF* V600E mutation test results using ARMS‐PCR and DPCR in FNA samples.

		ARMS‐PCR			*p*
		Positive	Negative	Total	
dPCR	Positive	26	2	28	*p* = 0.4795
	Negative	0	44	44	/
	Total	26	46	72	/

We noticed a drastic difference in DNA quantity used in these two test methods. All ARMS‐PCR tests were performed in our hospital's molecular pathology laboratory immediately following the FNA procedure and DNA extraction. The average DNA input for all 72 FNA tests was 170 ± 17.35 ng, while the average DNA input for the 28 positive *BRAF* V600E tests was 164.0 ± 12.0 ng. However, dPCR tests were performed using leftover genomic DNA from ARMS‐PCR tests that was stored in the −20°C freezer for up to 2 months, with an average DNA input of 14.9 ± 10.8 ng and 16.4 ± 9.7 ng for all 72 tests and the 28 positive *BRAF* V600E tests, respectively. Compared to ARMS‐PCR, the dPCR assay not only has higher sensitivity, but also requires much less DNA for a successful test. When pathology results were used as the standard, the sensitivity, specificity, PPV, and NPV of dPCR assay were all 100%, while those of ARMS‐PCR assay were 92.9% (26/28), 100% (44/44), 100% (26/26), and 95.7% (44/46), respectively (Table 4).

### 3.5 Validation of the Discrepant Samples With NGS

Given the LOD of our assay was set at 0.05%, we further validated three discrepant samples with a mutation frequency lower than this threshold. First, we serially diluted the DNA from RKO with DNA from PBMC from a healthy donor, with final concentrations of 0.01%, 0.04%, and 0.4%. We then performed NGS with barcoded primers for library construction to eliminate sequencing errors. The same DNAs were also used for dPCR. There is an excellent correlation between NGS and dPCR results (*R* ^2^ = 0.9994, Figure S2A), indicating that our NGS approach worked well. We then performed NGS using the three discrepant patient samples (15196, 00843, 01021) with low *BRAF* V600E mutation frequencies. Again, NGS results confirmed their mutation frequencies are consistent with dPCR results (Figure S2B).

## 4 Discussion

In this study, we developed a highly sensitive dPCR assay for the *BRAF* V600E mutation detection and evaluated its performance using PTC, cancer‐adjacent normal tissues, lymph nodes, and benign thyroid disease samples. As a relatively new technology, officially approved dPCR *BRAF* V600E mutation detection kits are not commercially available yet. Thus, ARMS‐PCR assays remain the gold standard in molecular pathology testing [14]. Our data clearly demonstrated that the performance of dPCR is superior to ARMS‐PCR in detecting the *BRAF* V600E mutation in pathological samples.

The presence of metastatic lymph nodes directly influences the surgical approach and postoperative treatment strategy of PTC patients [15]. However, lymph nodes with only psammoma bodies but without metastatic tumor cells present a great challenge for pathologists as they mostly rely on optical microscopes to make a diagnosis. Given that controversy remains over whether psammoma bodies can represent malignant tumors in the thyroid [16], a positive *BRAF* V600E test result would help to determine the presence of malignancy. In this study, we examined four central lymph node samples from PTC patients. With a highly sensitive dPCR assay, we detected a *BRAF* V600E mutation in one of the lymph nodes at an extremely low frequency of 0.04%, confirming the presence of micro‐metastasis. This patient should receive more aggressive treatment to eliminate residual metastatic disease or closer follow‐up for relapses following surgery. Our experience raised a very important, yet neglected question in clinic to date, that a lymph node with only psammoma bodies cannot be reliably determined to harbor micro‐metastasis by test methods commonly deployed in current clinical laboratories, including HE stain, IHC, and ARMS‐PCR, and more sensitive mutation detection methods, such as dPCR, should be used to minimize the likelihood of misdiagnosis [17].

Intraglandular dissemination of PTC is when cancer cells metastasize through lymphatic vessels within the gland and is the major cause of disease relapse following surgery [18]. PTC patients with intraglandular dissemination require more aggressive therapy or more frequent follow‐ups [19]. Classical pathology had difficulties identifying metastatic tumor clusters from normal thyroid tissue with a background of atypical follicular lesions, such as NIFTP or chronic lymphocytic thyroiditis, which also express the same tumor biomarkers as PTC does. In these scenarios, a reliable *BRAF* V600E test would be extremely valuable in helping make a diagnosis. In our studies, dPCR assays identified four “cancer‐adjacent” tissues with *BRAF* V600E mutations that were originally classified as *BRAF* WT by ARMS‐PCR. The extremely low proportion of tumor tissue in the sections, less than 0.1% of the tissue area, is likely the reason for the false‐negative ARMS‐PCR results. Indeed, these four specimens displayed very low mutation rates in our dPCR assays, ranging from 0.1% to 1.12%. Thus, our data clearly demonstrated that highly sensitive dPCR assays can improve PTC pathological diagnosis in atypical lesions and in samples with extremely low tumor proportions.

Another interesting finding of our study is dPCR's potential in *BRAF* V600E mutation detection for FNA samples. The volume of FNA biopsy is usually small in which multiple factors could determine the quantity of collectable cells, including puncture technique, distribution of blood vessels, tissue texture, and size and location of the tumor [20]. Failure of diagnosis following the FNA procedure is mostly due to insufficient number of collected cells and/or low quantity of genomic DNA extracted [21]. Our data clearly demonstrated that, compared to ARMS‐PCR, the extremely high sensitivity of dPCR makes it a better method to make a successful diagnosis that would minimize invasive biopsy injuries due to repeated FNA procedures.

For our assay to be practically useful, its cost needs to be considered. Since dPCR is a relatively new technology, it has not been as widely adopted in clinics as quantitative PCR (qPCR). In addition, each dPCR platform requires its own specific instrument and consumables, raising the cost of current dPCR assays. However, given experimental replicates are usually not required for dPCR assays and with a higher sensitivity, its clinical value, at least under certain application settings, will be gradually appreciated. With improved availability of more commercial assays and lowered costs for its consumables, we believe dPCR will eventually be as cost‐effective as qPCR in the future.

Our study has certain limitations. First, it's a retrospective single‐center study, including 180 FFPE samples and 72 FNA samples, with a small number of lymph node samples. The accuracy of our dPCR assay in detecting *BRAF* V600E mutation needs to be further validated in a multicenter study with a larger sample size. Second, the number of discrepant cases in our study is relatively small (*n* = 7); the clinical significance of this improved sensitivity will be clearer when long‐term outcome data of tested patients become available and such data are also included in future large multicenter studies.

Overall, our studies highlighted a clear advantage of dPCR over ARMS‐PCR in *BRAF* V600E detection in tissue specimens of PTC, and further study of dPCR's wider adoption in molecular pathological diagnosis is warranted.

## Funding

This work is supported by the National Natural Science Foundation of China (62203117) and the Clinical Research Fund of Huadong Hospital (HDLC2022012). This work is also funded by a Zhejiang Province Science and Technology Innovation Team program project (2021R02020) and a Yongjiang Talent Team project from the Department of Science and Technology of Ningbo (2023A‐094‐B).

## Ethics Statement

This study is approved by the Ethics Committee of Huadong Hospital (20240084).

## Conflicts of Interest

Ying Jiang, Liang Zheng, Xiu He, and Xin Huang are employees of Mission Medical Technologies.

## Supporting information


**Figure S1:** Development of the *BRAF* V600E mutation detection dPCR assay. A two‐dimension plot demonstrates the performance of the *BRAF* V600E mutation detection dPCR assay (left panel); Serial dilution assay at low concentrations of DNA inputs demonstrates a robust linear quantification ability of the dPCR assay (right panel).
**Figure S2:** NGS validation of the dPCR results. (A) Excellent correlation of NGS and dPCR results using serially diluted samples with 0.01%, 0.04%, and 0.4% *BRAF* V600E mutations (*R* ^2^ = 0.9994); (B) Correlation of NGS and dPCR results from patient samples 01021, 00843, and 15,196 (*R* ^2^ = 0.9926).

## Data Availability

The data that support the findings of this study are available from the corresponding author upon reasonable request.
