# Supplementary material for: Improving the Accuracy of Papillary Thyroid Cancer Pathological Evaluation With Digital PCR Detection of the BRAF V600E Mutation
Source: J Clin Lab Anal. 2026 Sep 16;40(19):e70323. doi: 10.1002/jcla.70323 (PMC13583474; doi:10.1002/jcla.70323)
Supplement: Supplementary file 1 [file JCLA-40-e70323-s001.docx]

**Supplemental Material**

**Improving the accuracy of papillary thyroid cancer pathological evaluation with digital PCR detection of the *BRAF* V600E mutation**

Na Yu^1*^, Kai Zhang^2*^, Ying Jiang^3^, Liang Zheng^3^, Xiu He^3^, Qinglin He^4^, Meng Dai^5^, Tianhua Hu^6^, Dongfang Tang^7^, Jingjing Jin^1^, Xin Huang^3, 7*^, Li Xiao^1*^

**Supplemental Materials and Methods**

**Tumor sample evaluation**

Surgical tissue samples were collected in the formalin solution and transferred to pathology laboratory, where they were processed, embedded, and sectioned according to standard pathology protocols. Each tumor was reviewed by two pathologists (N.Y. and L.X.) and tumor cell content was assessed by hematoxylin-eosin (HE) staining. DNA was prepared from four 5-μm-thick FFPE sections. DNA was extracted and purified with the QIAamp DNA FFPE Tissue Kit (Qiagen, Germantown, MD, USA). The FNA cytological samples were obtained through fine needle aspiration from the tumor and collected in sterile fixative. The samples were then immediately submitted to pathology laboratory where genomic DNA was extracted with the Nucleic Acid Extraction Kit (Rigen Biotechnology, Shanghai, China). Purified DNA was quantified with a NanoDrop 2000 spectrophotometer (ThermoFisher Scientific, Waltham, MA, USA). DNA samples were stored at 20 °C in Tris-EDTA buffer. And residual DNA samples after ARMS-PCR analysis were used for detection of *BRAF* V600E mutation by dPCR.

**Primer Design and Optimization**

The primers were designed to be around 20 bps in length using Primer3 software and their specificity was verified with UCSC Genome Browser Primer BLAT. All primers and probes were purchased from Shanghai Sangon Biotechnology. For each primer pair, we performed simplex PCR using human genomic control DNA and DNA from RKO, a colorectal cancer cell line with a *BRAF* V600E mutation, and confirmed the presence of the correct products with electrophoresis on a 2% agarose gel. After optimization of the primers, we selected one pair of primers and probes for our tests in this study as the following: BRAF-V600E-F AGACCTCACAGTAAAAATAGGTG, BRAF-V600E-R CCACAAAATGGATCCAGACAAC, BRAF-V600E-WT-probe HEX-TCGAGATTTCACTGTAGC-BHQ1, BRAF-V600E-MT-probe FAM-CGAGATTTCTCTGTAGCT-BHQ1.

**ARMS-PCR assay**

*BRAF* V600E mutation detection was performed using a commercially available ARMS-PCR kit (Rigen Biotechnology, Shanghai, China), according to the manufacturer’s instructions.

**dPCR assay**

Each amplification reaction was prepared in a final volume of 20μL containing 10μL of 2 X ddPCR Supermix for Probes (no dUTP) (Bio-Rad Laboratories, Hercules, CA, USA), 1.0–1.125 mol/L of primers, 0.312 mol/L of probes. We used a variable amount of input DNA that ranged from 1 to 30 ng, according to NanoDrop readings. The thermocycling protocol consisted of an initial denaturalization step of 10 min at 95 °C, followed by 39 cycles of 30 s at 94 °C and 1 min at 60 °C plus a final incubation time of 10 min at 98 °C. Droplets were analyzed in an automated QX200 droplet reader (Bio-Rad Laboratories, Hercules, CA, USA). The fractional abundance of each allele, expressed as variant allele frequency (VAF), was calculated using the QuantaSoft ver 1.7.4 software (Bio-Rad Laboratories, Hercules, CA, USA).

**NGS and data analysis**

Sequencing library was constructed with two rounds of PCR. In the first round, randomly barcoded primers were used to amplify the *BRAF* locus for 30 cycles. The first round PCR product was cleaned and eluted into 20μl of water and 1μl of the cleaned DNA was used for the second round of PCR. The second round of PCR was performed for 25 cycles to add sequencing primers and indexes. The library was sequenced on a DNBSEQ-T7 platform (MGI Tech, Shenzhen, China). The following primers were used for library construction: BRAF-NGS-Fw: CAGCGTCAGATGTGTATAAGAGACAGCATGAAGACCTCACAGTAAA

BRAF-NGS-Rev: GGCTCGGAGATGTGTATAAGAGACAGNNNNNNNNNNCAAAATGGATCCAGACAAC

Second round Fw: AATGATACGGCGACCACCGAGATCTACAC(i5_index)TCGTCGGCAGCGTCAGATGTGTATAAGAGACAG

Second round Rev: CAAGCAGAAGACGGCATACGAGAT(i7_index)GTCTCGTGGGCTCGGAGATGTGTATAAGAGACAG

Raw sequencing data were filtered using fastp (v0.23.4) to obtain high-quality reads with the parameters: --detect_adapter_for_pe -n 10 -e 20 -l 100 -q 15 -u 40 -p. The filtered reads were then aligned to the human GRCh38 (hg38) reference genome using the BWA-MEM algorithm (v0.7.18) with parameters set as -t 8 -k 19 -T 30 -L 20 -Y. The aligned files were sorted using SAMtools (v1.20), and the counts of the four nucleotides at the *BRAF* V600E mutation locus were subsequently quantified using IGV (v2.12.3).

**Statistical analysis**

The statistical analyses were performed using the SPSS version 19.0 software. Continuous data are presented as means ± standard deviation. Categorical variables were reported as frequencies and percentages. McNemar's test was used for paired categorical data. A p value of less than 0.05 was considered to be significant.

*The sensitivity, specificity,* *positive predictive value (PPV), and negative predictive value (NPV) were calculated according to standard statistical formulas using histopathological results as a standard to evaluate the performance of ARMS-PCR and dPCR assays. The 95% confidence intervals (CIs) for sensitivity, specificity, PPV, and NPV of ARMS-PCR and dPCR assays were calculated using the Wilson Score interval method as implemented in R’s prop.test function.*

**Supplementary Figure1.** Development of the *BRAF* V600E mutation detection dPCR assay. A two-dimension plot demonstrates the performance of the *BRAF* V600E mutation detection dPCR assay (left panel); Serial dilution assay at low concentrations of DNA inputs demonstrates a robust linear quantification ability of the dPCR assay (right panel).

**Supplementary Figure 2.** NGS validation of the dPCR results. A. Excellent correlation of NGS and dPCR results using serially diluted samples with 0.01%, 0.04%, and 0.4% *BRAF* V600E mutations (R^2^=0.9994); B. Correlation of NGS and dPCR results from patient samples 01021, 00843, and 15196 (R^2^=0.9926).
